# Skin Hyperpigmentation: An Under‐Recognized Dermatological Clue to Vitamin B12 Deficiency

**DOI:** 10.1002/ccr3.72634

**Published:** 2026-04-27

**Authors:** Mahesh Mathur, Sumit Paudel, Sambidha Karki, Sandhya Regmi, Shilpa Maharjan, Nabita Bhattarai

**Affiliations:** ^1^ Department of Dermatology College of Medical Sciences Bharatpur Nepal

**Keywords:** cutaneous hyperpigmentation, hyper‐segmented neutrophils, nutritional anemia, reversible skin pigmentation, vitamin B12 deficiency

## Abstract

Cutaneous hyperpigmentation, an overlooked manifestation, can be an early sign of vitamin B12 deficiency. This case highlights symmetric pigmentation on the dorsum of hands and palms in a long‐term vegetarian. Recognizing such subtle skin changes aids early diagnosis. Timely treatment reverses both pigmentation and systemic symptoms, underscoring its clinical relevance.

## Graphical abstract

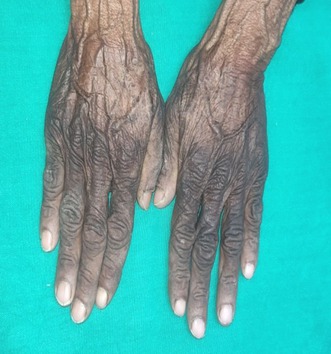

**Abbreviations**
MCV: mean corpuscular volume
MSH: melanocyte stimulating hormone
PBS: peripheral blood smear
RDA: recommended daily allowance

## 1 Introduction

Vitamin B12 is a water‐soluble vitamin, synthesized only by microorganisms. The dietary source of vitamin B12 is food of animal origin like meat, fish, and dairy products [1]. Vitamin B12 deficiency is a common cause of systemic manifestations including megaloblastic anemia, pancytopenia and cutaneous manifestations [2].

Prevalence of vitamin B12 deficiency varies from 3% to 5% in the general population and increase from 5% to 20% among people older than 65 years [3]. 10% of vitamin B12 deficient patients show reversible skin pigmentation [1]. Common cause of vitamin B12 deficiency include malabsorption, due to pernicious anemia, gastric and ileum resection, celiac disease, inadequate intake in vegetarian group of population, diphyllobothrium latum infection, pancreatic insufficiency and intake of interfering medications such as metformin or proton pump inhibitors [1, 4]. Herein, we present a case of cutaneous and neurological manifestations in patient with vitamin B12 deficiency that can be the clue for the early diagnosis.

## 2 Case History and Examination

**FIGURE 1:**
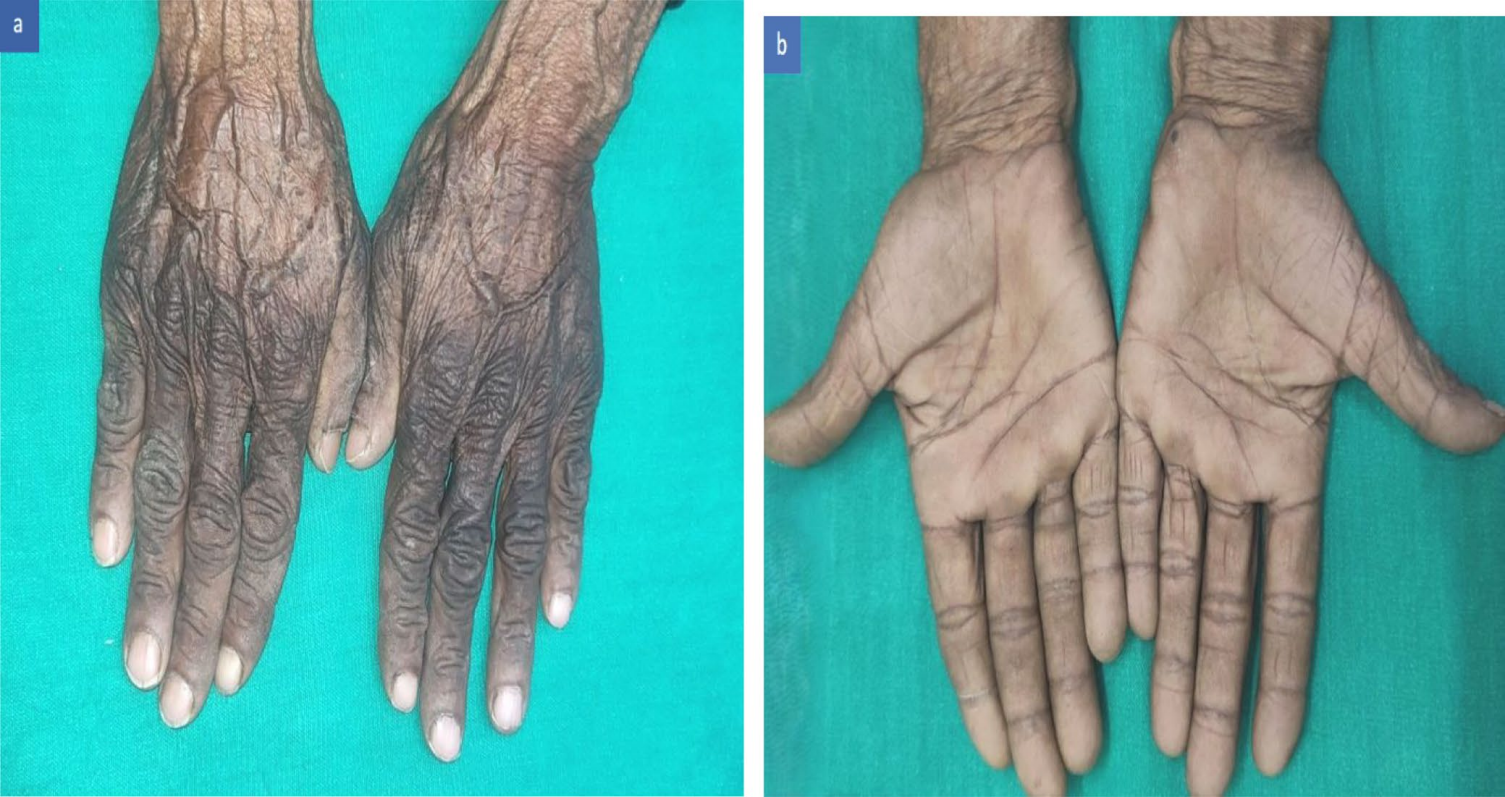
Before treatment: (a) Hyperpigmentation over the dorsal aspect of both hands, prominently involving the knuckles, (b) Hyperpigmentation over the palmar aspect, accentuated along the palmar creases.

A 69 years male with Fitzpatrick skin type IV presented with asymptomatic hyperpigmentation over dorsum of hand bilaterally and symmetrically distributed for 2 months. Lesions initially started at knuckles of hands which gradually progressed to involve bilateral dorsal aspect of hands including periungal regions (Figure 1a), palmar aspect of bilateral hands including palmar creases (Figure 1b). The lesions were diffuse, ill‐defined to moderately well defined with brown to dark brown coloration and smooth surface without scaling. There was no mucosal hyperpigmentation in the oral cavity, tongue, or lips, no evidence of glossitis or angular cheilitis. Nails were normal with no melanonychia and hair showed no abnormalities or depigmentation. He also complained of tingling sensation over bilateral foot, suggestive of early peripheral neuropathy, significant weight loss and easy fatigability for past 6 months. Patient consumes vegetarian diet for last 20 years. He applied various over the counter topical medications in skin lesions but no improvement was noticed. The patient did not report symptoms associated with adrenal insufficiency, such as postural dizziness, hypotension, salt craving, or gastrointestinal complaints like nausea, vomiting, abdominal pain.

## 3 Investigations

Lab investigations revealed slightly low hemoglobin concentration (11.1 g/dL, normal: 13–17 g/dL). Mean corpuscular volume (MCV) was in the upper normal range (96 fL, normal: 83–98 fL). Red cell distribution width (14%, normal: 11%–14%), white blood cells 5.56 × 10^9^/L (4–11 × 10^9^/L), neutrophils 70%, lymphocytes 25%, eosinophils 01%, monocytes 04%, and platelets 151 × 10^9^/L (150–450 × 10^9^/L) were reported.

**FIGURE 2:**
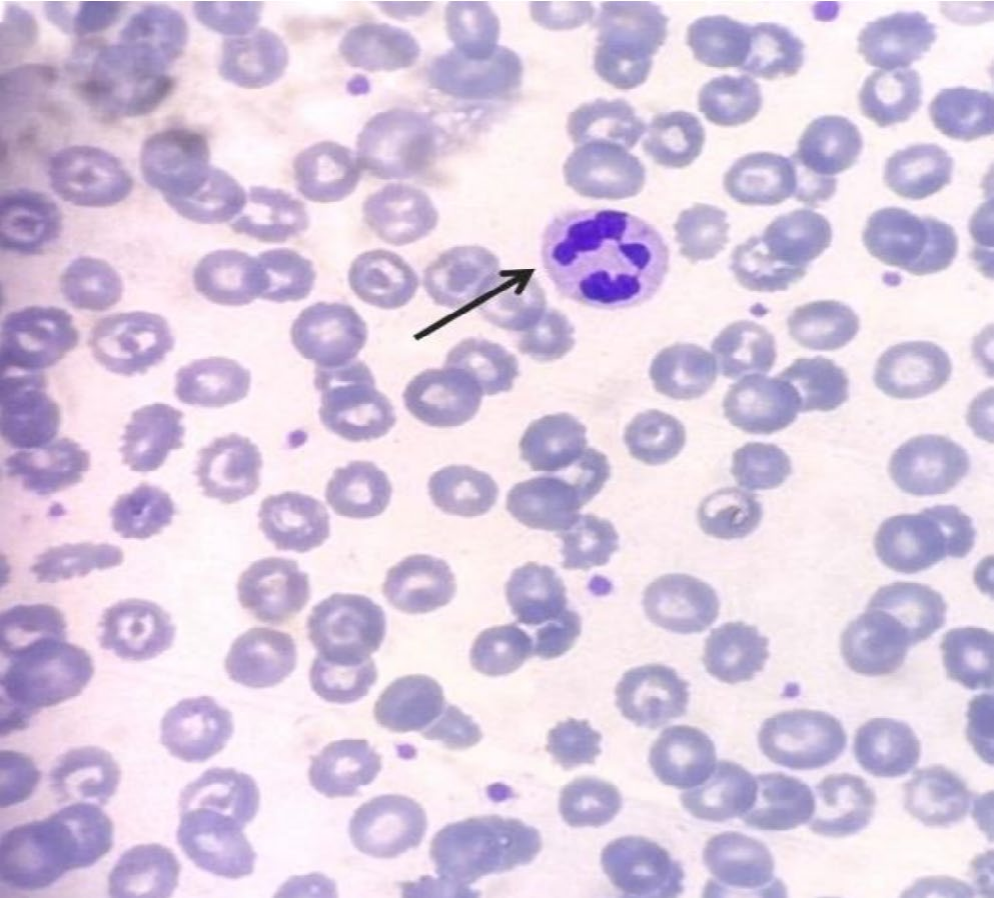
Hyper‐segmented neutrophils in vitamin B12 deficiency.

Serum vitamin B12 level was reduced (132 pg/mL; reference range 197–771 pg/mL) and peripheral blood smear showed normocytic anemia and hyper‐segmented neutrophils (6 lobes) with no schistocytes or spherocytes observed (Figure 2). Serum folate level and other lab investigations (serum electrolytes, liver and renal function tests) were within normal limits.

## 4 Treatment

Diagnosis of vitamin B12 deficiency manifesting as cutaneous pigmentation was made. He was treated with intramuscular vitamin B12 (cyanocobalamin) injection 1000 mcg daily for 10 days, then oral supplementation with a 1500 mcg daily dose for 3 months.

## 5 Outcome and Follow Up

**FIGURE 3:**
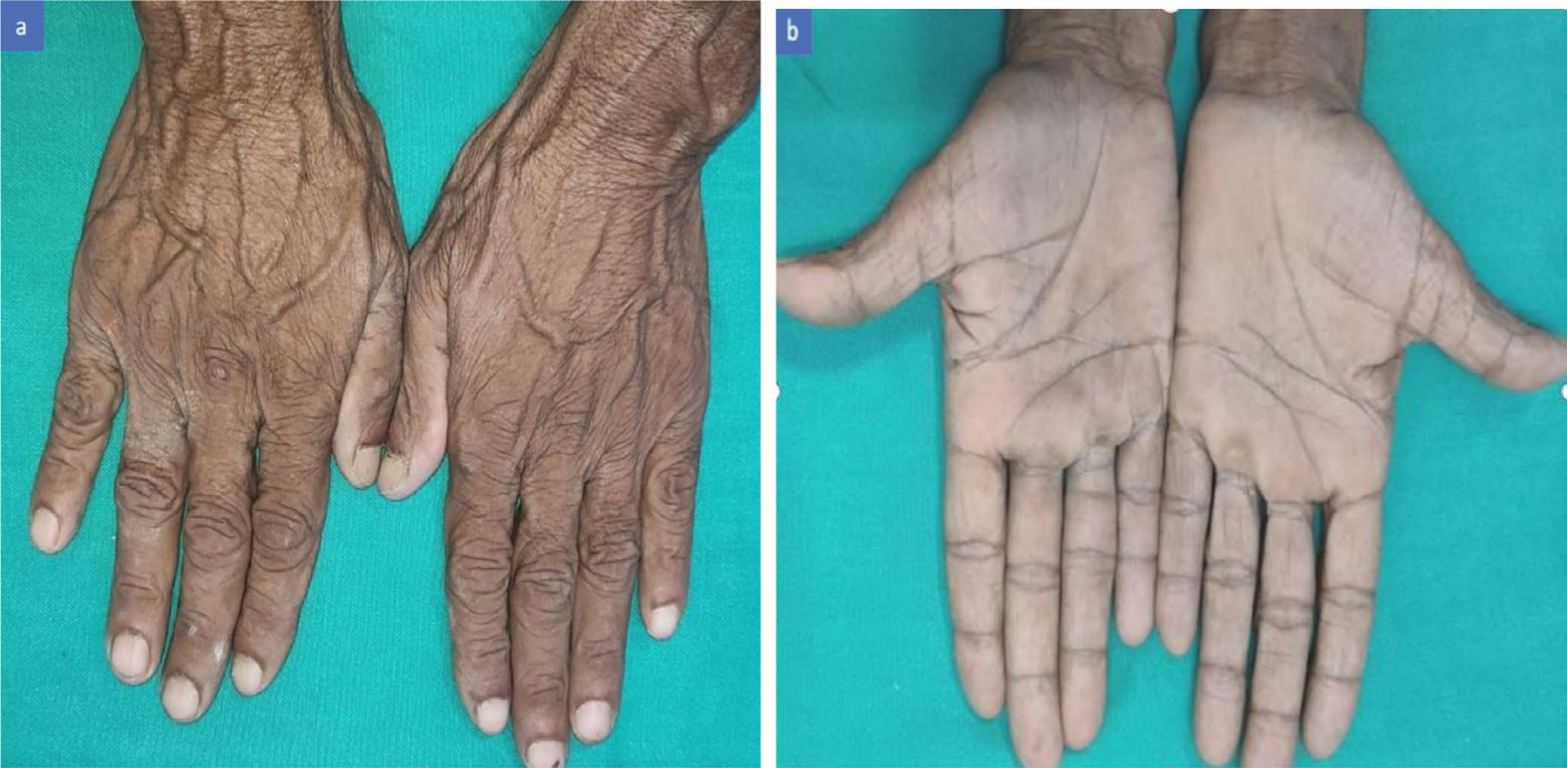
(a, b) Significant improvement in hyperpigmentation over the dorsal and palmar aspects after 3 months of treatment.

Serum vitamin B12 levels raised to 256 pg/mL at 3 months follow up. Neurological symptoms (tingling in feet) improved within 4–6 weeks. Cutaneous hyperpigmentation showed marked improvement by 3 months (Figure 3a,b). Fatigability and constitutional symptoms also gradually improved by 3 months. Maintenance therapy was further advised for next 3 months and repeat serum vitamin B12 level was planned. Despite this recommendation, the patient lost follow‐up, as he experienced significant symptomatic improvement by first three‐month visit and faced challenges in traveling to the hospital for further appointments.

## 6 Discussion

Vitamin B12 exists in various forms like cyanocobalamin, hydroxocobalamin, and methylcobalamin with a cobalt atom at the center of a corrin ring [1]. It is stored in the liver which may last for up to 5–10 years before clinical manifestations of vitamin B12 deficiency occur. It is first bound within the duodenum and jejunum to intrinsic factor produced by gastric parietal cells and is then absorbed in the terminal ileum [4].

The recommended daily allowance (RDA) of vitamin B12 is 2.4 mcg daily for 18 years and older. While in pregnant and breastfeeding, the RDA is 2.6 mcg and 2.8 mcg respectively. In children, it varies according to age [5]. Vitamin B12 deficiency is associated with neurologic, gastrointestinal, dermatologic and cardiovascular manifestations (Table 1) [6]. Deficiency is considered to be mild if its plasma concentration is 200–350 pg/mL, moderate if between 100 and 200 pg/mL and severe if between 50 and 100 pg/mL [7]. Our patient had moderate vitamin B12 deficiency.

**TABLE 1:** Manifestations of vitamin B12 deficiency.

Systems involved in vitamin B12 deficiency	Clinical manifestations
Dermatologic	Hyperpigmentation of the skin, palm, interphalangeal joints, terminal phalanges of the soles of the feet, and oral mucosal membrane, hair and nail changes, glossitis, angular stomatitis, and vitiligo
Anemia	Diminished energy and exercise tolerance, fatigue, shortness of breath, and palpitations
Neuropsychiatric	Tingling and numbness, abnormalities of gait, loss of concentration to memory loss, disorientation
Gastro‐intestinal	Sore tongue, appetite loss, flatulence, and constipation
Cardiovascular	Atherosclerosis, stroke and infarction

The pathogenesis of hyperpigmentation in vitamin B12 deficiency involves a decrease in the reduced glutathione level and increased tyrosinase activity, resulting in increased melanin synthesis. A defect in melanin transfer between melanocytes and keratinocytes and increased biopterin in vitamin B12 deficiency increasing hydroxylated phenylalanine have also been considered in the pathogenesis of hyperpigmentation in vitamin B12 deficieny [8].

Macrocytic anemia is typically seen in vitamin B12 deficiency. The diagnosis of vitamin B12 deficiency is supported by low serum cobalamin levels, along with elevated methylmalonic acid and homocysteine levels. In some cases, testing for intrinsic factor and parietal cell antibodies helps to identify pernicious anemia as an underlying cause [9].

In our case, other potential causes including pernicious anemia, gastrointestinal malabsorption, and chronic medication use were also considered. However, the patient denied any gastrointestinal complaints, and a brief medication history did not reveal any agents known to interfere with vitamin B12 absorption. Antibodies to intrinsic factor and parietal cell antibodies were not performed due to economic constraints. Serum methylmalonic acid and homocysteine, which are sensitive indicators of vitamin B12 deficiency, are not available in our setting but are recommended in diagnostic evaluation. Iron studies were not performed, but the near‐normal MCV indicates coexistent iron or other nutrient deficiencies. Considering these factors in a patient with a long‐term vegetarian diet and the excellent clinical response to vitamin B12 supplementation, dietary deficiency remains the most likely cause.

Differential diagnosis includes chronic primary adrenal insufficiency, where elevated melanocyte stimulating hormone (MSH) levels lead to increased melanin synthesis, folate deficiency in which symptoms overlap with vitamin B12 deficiency except for the classical neurological features, which are less likely to be present in folate deficiency [9, 10].

Adrenal insufficiency should be considered, particularly in cases of generalized pigmentation involving sun‐exposed areas, palmar creases, and mucosal involvement. Diagnostic evaluation includes measurement of early morning serum cortisol and adrenocorticotropic hormone levels, with confirmatory testing such as the ACTH stimulation test [11]. Morning serum cortisol and plasma ACTH levels were not performed due to resource limitations. However, the absence of clinical signs of adrenal insufficiency makes Addison's disease unlikely in our patient.

Autoimmune disorders, including connective tissue diseases, may also present with pigmentary changes and should be evaluated using appropriate clinical correlation and serological markers such as antinuclear antibodies [12].

Furthermore, HIV infection has been associated with mucocutaneous hyperpigmentation, either due to the infection itself or secondary to medications, and should be ruled out if clinically indicated [13].

In regions where tuberculosis is prevalent, it should be considered as a potential underlying cause, especially when the patient is taking antitubercular medications (rifampicin and isoniazid). Investigations include chest imaging and microbiological tests [14].

In view of dietary history, progressive cutaneous hyperpigmentation, associated constitutional symptoms (easy fatigability, weight loss), and neurological complaints (tingling sensation over bilateral feet), laboratory investigations were performed. Low serum vitamin B12 level, MCV more than 90 in an appropriate clinical setting and hyper‐segmented neutrophils in peripheral blood smear (PBS) confirmed the diagnosis of vitamin B12 deficiency in our case. The diagnosis was further supported by an excellent clinical response to vitamin B12 supplementation.

Treatment consists of oral and/or parenteral vitamin B12 depending on the severity of symptoms and the level of deficiency. If cutaneous hyperpigmentation is the primary presentation, oral treatment is preferred over parenteral treatment. The recovery period of the hyperpigmentation tends to vary from 6 to 12 weeks after the treatment [15]. Our patient had moderate vitamin B12 deficiency along with neurological features, so we started intramuscular vitamin B12 injection for 10 days followed by oral supplementation for 3 months.

Nottinghamshire guidelines [16] recommend maintenance with intramuscular hydroxocobalamin 1 mg every 3 months. However, we gave oral methylcobalamin 1500 mcg (which is available in our settings) daily for 3 months in this patient to enhance compliance and facilitate home‐based therapy.

At 3 months follow‐up, serum vitamin B12 level improved from 132 pg/mL to 256 pg/mL, indicating a significant response to therapy. Hematologically, there was improvement in hemoglobin levels. In addition to cutaneous improvement, the patient reported marked reduction in fatigability and improvement in tingling sensation in the lower limbs. These findings support a strong association between vitamin B12 deficiency and the observed clinical manifestations.

Vitamin B12 deficiency may present with neurological manifestations such as paresthesia, numbness, gait disturbances, and cognitive changes. Early recognition of these symptoms is crucial, as neurological deficits may become irreversible if treatment is delayed [17]. In our case, the presence of tingling sensation over the lower limbs served as an important early clinical clue supporting the diagnosis.

This case is reported as cutaneous hyperpigmentation following the vitamin B12 deficiency has rarely been mentioned in the literature. Our case report highlights skin as an important diagnostic window into systemic disease following the nutrition deficiency.

## 7 Summary

Cutaneous manifestation due to vitamin B12 deficiency is often overlooked in the early stage of the disease but it can be the earliest reversible manifestation of vitamin B12 deficiency. Early detection and adequate treatment will prevent anemia and various neurological manifestations.

## Author Contributions

**Mahesh Mathur:** conceptualization, methodology, supervision, visualization. **Sumit Paudel:** data curation, formal analysis, investigation, software. **Sambidha Karki:** conceptualization, supervision, validation, visualization. **Sandhya Regmi:** data curation, methodology, validation, visualization. **Shilpa Maharjan:** conceptualization, formal analysis, visualization. **Nabita Bhattarai:** conceptualization, investigation, methodology, visualization, writing – original draft.

## Funding

The authors have nothing to report.

## Disclosure

Prior Publication: This material has not been published previously.

## Ethics Statement

Reviewed and approved by Institutional review board College of medical sciences (IRBCOMS). The patients in this manuscript have given written informed consent to the publication of their case details.

## Consent

The authors obtained written consent from the patient for use of photographs and medical information to be published online and with the understanding that this information may be publicly available and discoverable via search engines. Patient consent forms are not provided to the journal but are retained by the authors.

## Conflicts of Interest

The authors declare no conflicts of interest.

## Data Availability

The data that support the findings of this study are available from the corresponding author upon reasonable request.
